# Quantitative determination of the electric field strength in a plasmon focus from ponderomotive energy shifts

**DOI:** 10.1515/nanoph-2022-0284

**Published:** 2022-08-02

**Authors:** Pascal Dreher, David Janoschka, Alexander Neuhaus, Bettina Frank, Harald Giessen, Michael Horn-von Hoegen, Frank-J. Meyer zu Heringdorf

**Affiliations:** Faculty of Physics and Center for Nanointegration, Duisburg-Essen (CENIDE), University of Duisburg-Essen, 47048 Duisburg, Germany; 4th Physics Institute, Research Center SCoPE, and Integrated Quantum Science and Technology Center, University of Stuttgart, 70569 Stuttgart, Germany; Interdisciplinary Center for the Analytics on the Nanoscale (ICAN), 47057 Duisburg, Germany

**Keywords:** photoelectron spectroscopy, photoemission microscopy, ponderomotive energy, surface plasmon polariton

## Abstract

Spectroscopic photoemission microscopy is used to detect and quantify a ponderomotive shift in the energy of electrons that are emitted from a surface plasmon polariton focus. The focus is formed on an atomically flat Au(111) surface by an Archimedean spiral and is spatiotemporally separated from the circularly polarized light pulse used to excite the spiral. A spectroscopic analysis of electrons emitted from the focus exhibits a peaked above-threshold electron emission spectrum. From the shift of the peaks as function of laser power the field strength of the surface plasmon polariton was quantitatively determined *without* free parameters. Estimations of the Keldysh parameter ***γ*** = 4.4 and the adiabaticity parameter ***δ*** = 4700 indicate that electron emission occurs in a regime of multiplasmon absorption and nonlocalized surface plasmon fields.

## 1 Introduction

Nano-optics aims at controlling optical fields at the nanoscale to achieve novel functionality [1, 2]. For instance, field-enhancements in nano-optical systems can create strong-field situations and enable high-harmonic generation [3, 4], photoinduced near-field electron micropscopy [5–8], field-enhanced Raman spectroscopy [9], and other nonlinear light–matter interactions [10–12].

When creating strong fields by focusing of intense lasers, one encounters the fundamental problem of the diffraction limit: the attainable focus dimension of a Gaussian laser beam depends on the beam-diameter at the position of the focusing lens, its focal length, and on the wavelength of the light. The diffraction limit, however, can be bypassed by converting laser pulses into shorter wavelength surface plasmon polaritons (SPPs), and by focusing the SPPs rather than the laser pulses. It was demonstrated that converting 800 nm wavelength laser pulses into short-range SPPs of 180 nm wavelength at a gold–silicon interface resulted in a nonlinear photoelectron emission spot of only 60 nm diameter [13]. Understanding the nonlinear electron emission from such a focus requires detailed knowledge of the local SPP field, i.e., the field orientation and field strength. While the orientation of SPP field vectors can be measured using near-field microscopy techniques [14, 15] or – with sub-femtosecond time-resolution – by employing the recently developed technique of vector microscopy [16], a quantitative measurement of the absolute field strength in an SPP focus is a challenging endeavor that has not been resolved yet.

In the present work we measure the ponderomotive energetic shift of electrons emitted from a flat Au(111) surface in an intense SPP focus that is spatiotemporally separated from the exciting laser pulse. The ponderomotive energetic shift is used to *quantitatively* derive the local SPP field strength in the focus without free parameters.

## 2 Methods

The experiment is based on the experimental setup around the spectroscopic photoemission and low energy electron microscope [17] (ELMITEC SPE-LEEM III) at the University of Duisburg-Essen. This microscope has been combined with a <15fs -pulsed Ti:Sapphire laser to enable nonlinear photoemission microscopy (PEEM) in a normal-incidence geometry [18] and is equipped with a single-electron sensitive imaging CMOS detector [19]. Mutually delayed laser pulses are created by a Pancharatnam’s phase stabilized Mach–Zehnder interferometer [20, 21] that can be bypassed for single-pulse experiments.

For the present study we use self-assembled Au platelets that were synthesized *ex-situ* by a single step thermolysis of (AuCl_4_)^−^-tetraoctylammonium bromide [22]. A focused ion beam (Raith ionLINE Plus) was used to mill grooves into the surface that provide momentum matching and enable conversion of the laser pulses into SPPs. After fabrication of the grooves, the sample was placed in the load-lock system of the SPE-LEEM, plasma-cleaned, and directly transferred into the ultrahigh-vacuum preparation chamber where several cycles of standard Ar-ion sputtering and annealing were employed.

**Figure 1:**
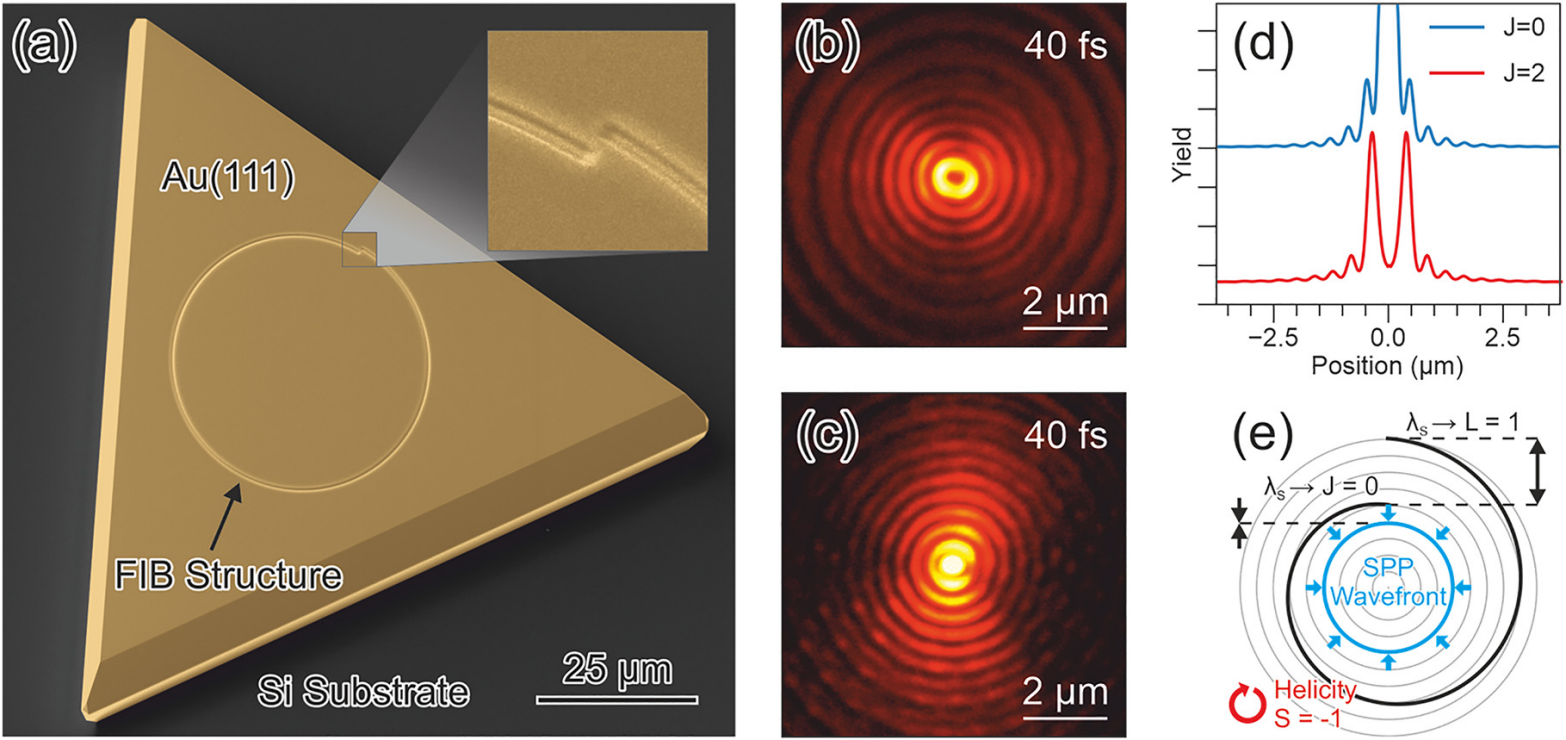
(a) Au island with an Archimedean spiral milled into it. The inset shows the gap of one SPP wavelength (*L* = 1). (b) Logarithmically scaled PEEM image of the center of the spiral for excitation with *S* = 1 circularly polarized light yielding a “doughnut” mode with *J* = 2. (c) Logarithmically scaled PEEM image for excitation with *S* = −1 circularly polarized light yielding a focus spot in the center of the spiral with *J* = 0. (d) Azimuthally-averaged photoemission yield extracted from panels (b) and (c) in linear scaling showing the strong enhancement of the emission in the center (*J* = 0, blue) and in the ring comprising the doughnut (*J* = 2, red). The curves are vertically shifted with respect to each other. (e) Illustration of the focus formation. As the field orientation in the surface plane rotates clockwise within each optical cycle, SPPs are excited at different locations along the groove. For *S* = −1 helicity, SPPs excited at earlier times propagate longer to the center of the spiral. In the center all SPPs interfere constructively.

In the following we will first describe the concept how to form a SPP focus and then create strong SPP fields and quantify their field strength. Figure 1(a) shows an example of a self-organized Au island with a groove in the shape of an Archimedean spiral. The radius *r* of the spiral increases as function of the polar angle *φ* as $r\left(\varphi \right)=r_{0}+L\cdot \frac{\varphi }{2\pi }\lambda_{s}$ , i.e., after a full revolution *φ* = 2*π* the radius has increased by *L* SPP wavelengths *λ*_*S*_. Accordingly, for a spiral with *L* = +1 that is comprised of only one revolution, a discontinuity of *λ*_*S*_ is formed like the one shown (enlarged) in the inset of Figure 1(a).

## 3 Results and Discussion

It is known that Archimedean spirals that are illuminated with circularly polarized light can be used for optical spin to orbital angular momentum conversion [23, 24] of light and SPPs. The total angular momentum of the excited SPP arises from the topological charge *L* of the spiral and the spin angular momentum *S* of the exciting light. Nonlinear PEEM images from a time-resolved experiment at a pump-probe-delay of Δ*t* = 40 fs for excitation with circularly polarized light of different handedness are shown in a logarithmic scale in Figure 1(b) and (c), reflecting angular momentum states of *J* = 2 and *J* = 0, respectively. Please note that for recording these panels the work function of the Au surface was lowered by Cs deposition from a standard Cs dispenser (SAES Getters) to enable a second-order photo electron emission process [25].

The second-order electron yield in the nonlinear PEEM images of Figure 1(b) and (c) is described by a time-integral of the fourth power of the sum of all time-dependent electric fields at the surface [26]. The SPP pulse is excited at the groove and propagates across the surface while the laser field homogeneously illuminates the surface. As a consequence the laser and SPP fields do not spatiotemporally overlap everywhere, and the spatial distribution of the detected electron yield is caused by a spatiotemporally dependent mixing of the SPP and laser fields. To acknowledge this complication, the terminology of plasmoemission was introduced to describe electron emission that occurs exclusively from the SPP’s electric field [27]. Since electrons emitted by plasmoemission do not require a probing laser pulse, plasmoemission does exhibit a particular delay-time-signature [27] and can in a pump-probe experiment easily be distinguished from the pump-probe contrast.

In Figure 1(b) and (c) the signatures of the plasmoemission are concentric circles, spaced by half the SPP wavelength, whereas the pump-probe contrast provides a direct conceptual visualization of the SPP [18] as projected onto the probing laser pulse, and shows spiral-shaped wave-fronts reflecting the *L* = 1 shape of the spiral [24, 28]. The overall dominance of the plasmoemission signature is further illustrated by the azimuthally averaged sections through panels (b) and (c) that are plotted in Figure 1(d).

In plasmoemission, Figure 1(b) exhibits a doughnut-type intensity distribution described by a Bessel mode $J_{2}\left(r\right)$ while the pattern in Figure 1(c) exhibits a *J*_0_(*r*) Bessel mode with a maximum in the center [29]. In the present work only the plasmoemission contribution to the electron yield is relevant, and below we will use the *J*_0_(*r*) Bessel mode to create a strong SPP focus. The formation of the focus can be easily understood with the sketch in Figure 1(e). Here, the field orientation of the circularly polarized laser pulse is assumed to rotate clockwise in the surface plane (*S* = −1). Accordingly, within each optical cycle SPPs are launched at different times at different locations along the groove. As time progresses, the position, where launching occurs, is closer to the center of the spiral. The later excitation time in each optical cycle and the shorter distance to the center are exactly balanced out for *L* = 1 and *S* = −1, with the consequence that all phase fronts arrive in the center at the same time and with the same phase. The resulting interference field is a standing SPP wave where the field in the $J_{0}\left(r\right)$ focus is oriented perpendicular to the surface.

**Figure 2:**
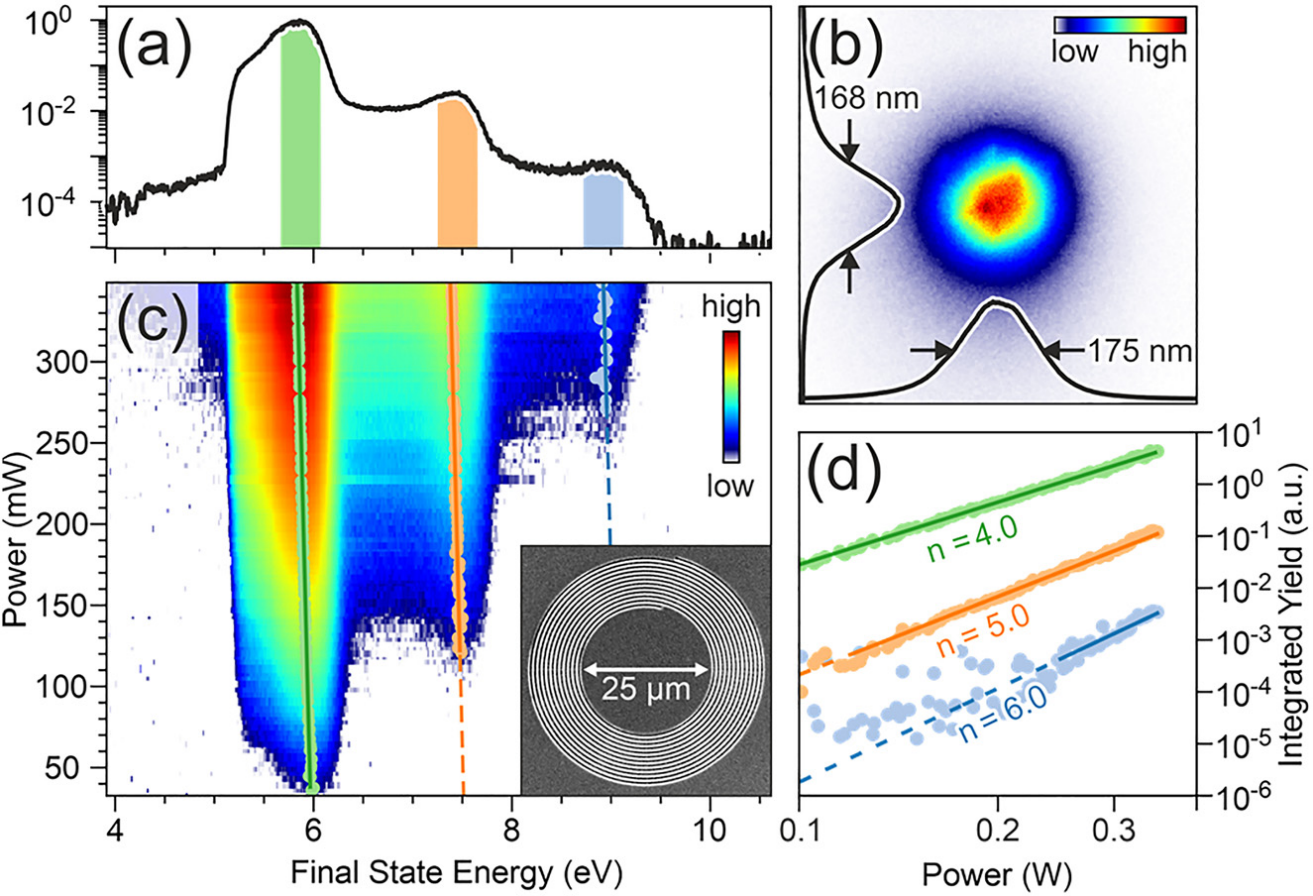
(a) Electron emission spectrum from the focus in the center of the spiral at an average laser power of 349 mW. The colored regions indicate the integration areas analyzed in panel (d). (b) PEEM image of the focus in the center of the spiral. The corresponding marginal distributions in x- and y-direction are plotted as red curves. (c) Logarithmic false-color representation of the energy-resolved electron emission yield as function of average laser power. The inset in panel (c) shows a scanning electron micrograph of the used spiral. (d) The yield-scaling of the emission peaks in panel (a) with the average laser power. The solid lines are power-law fits to the data with the indicated exponents.

To achieve a strong SPP field we are now going to apply the discussed concept to an *L* = 1 spiral with 12 revolutions (see scanning electron microscopy image in the inset in Figure 2(c)) that is also excited with *S* = −1 light to form a $J_{0}\left(r\right)$ focus spot. Also, rather than performing a time-resolved pump-probe experiment, we now only work with single pulses, thus suppressing all pump-probe contrasts. Note that the large number of revolutions in the grating coupler makes the SPP pulses significantly longer ( ≈30fs ) than the exciting laser pulses [30].

Figure 2(b) shows a close-up of the emission focus in the center of the structure. Marginal distributions of the electron yield from the focus in horizontal and vertical direction are shown as insets. The full width at half maximum of the focus spot of ≈170nm is well below the diffraction limit for *λ*_*S*_ ≈ 780 nm SPPs and is already indicative of the nonlinear character of the electron emission process. With a spiral radius of *r*_0_ = 12.5 μm and a SPP group velocity of 93.6% of the speed of light in vacuum [31] the SPP propagates for 45 fs to the focus, which implies that at the time the focus is formed, the <15fs short exciting laser pulse has already left the surface and does not contribute to the field strength at the time of electron emission. As such, the electron yield is entirely caused by plasmoemission.

The electron emission spectrum in Figure 2(a) was obtained by placing an aperture in a conjugate image plane [17] of the microscope to record only electrons that are emitted from the focus of the spiral. The spectrum shows several distinct peaks and plateaus. Plotting the intensity of the marked individual peaks as a function of the laser power in a double-logarithmic plot in Figure 2(d) shows that electrons are emitted by 4th, 5th, and 6th order processes. Note that in this experiment the sample was not cesiated and that all emission processes below fourth order were suppressed due to the work function of 5.3 ± 0.1 eV [32] of the clean Au(111) surface. A rough estimate of the electron count at the detector, based on its quantum efficiency, results in a maximal overall electron count of less than one electron per laser pulse. The peaks in the spectrum of Figure 2(a) are explained by emission from the Au surface state in different emission orders (see Supplementary Material), and the drop-offs at higher final-state energies arise from replica of the Fermi edge in the different emission orders.

Figure 2(c) shows a false-color representation of the energetically resolved emission yield as function of average laser power. Below a power of 100 mW only a 4th order process is visible. At about 120 mW a channel with 5th order emission becomes feasible, and at powers above 250 mW the 6th order emission sets in. Note that spectral broadening due to Coulomb repulsion (space charge effects) is below our experimental energy resolution. The maxima of the emission peaks (indicated by red, orange and blue dots) for the different emission orders show a systematic linear shift of all spectral features to lower final state energies with increasing laser power. The shifts for all emission orders are fitted by the same linear function with a slope of −425 ± 13 meV/W.

At first such a laser-power-dependent loss of kinetic energy seems to be in contradiction to results obtained for SPP field enhancements at sharp nano-tips [33–35] or prisms [36–39], where kinetic energy gains on the order of keV have been observed. These experiments are commonly explained by strong acceleration of the liberated photoelectrons by the intense oscillating near-field gradients at the surface. We will argue, however, that the particular combination of SPP field strength, field localization, and electron energies in the present work results in an adiabatic energy exchange between the emitted electrons and the driving electromagnetic fields. As such, the emission regime is different [10] and the interpretation is not unlike the one discussed for atomic physics [40, 41] where similar kinetic energy losses have been reported.

**Figure 3:**
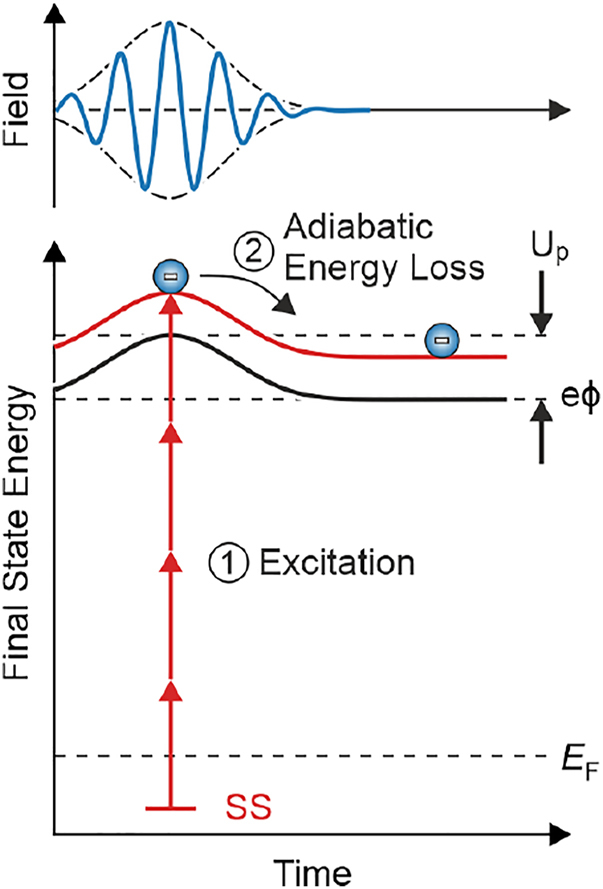
Energy-time diagram of the ponderomotive interaction in the adiabatic limit for 4th order electron emission. An electron is emitted at the maximum of the field envelope (step 1). As time progresses, the field envelope decays and the electron adiabatically loses the ponderomotive energy *U*_p_, resulting in a down-shift of the electron’s final-state energy (step 2).

We explain the observed linear shift by a ponderomotive interaction as illustrated for 4th order emission in Figure 3. In the limit of a weak SPP field an electron in the surface state (SS) can overcome the work-function *e* *ϕ* and can be excited into a free-electron state via a fourth-order process. However, due to the presence of the strong time-dependent SPP field (see light blue curve in Figure 3), the final state of the emission process is not a free electron state but instead needs to include the coupling between the emitted electron and the strong SPP field [42]. This coupling causes the electron to perform a quiver motion directly after photoexcitation, where the quiver movement is oriented along the surface normal component of the SPP field. In our case, as will be discussed in detail below, the emitted electron has an energy close to the vacuum level and it does not significantly propagate away from the surface within each oscillation period of the SPP field. It is thus sufficient to consider the coupling of the electron and the SPP field after emission in a picture where the fast oscillations of the field, and thus the fast quiver motion of the electron, are averaged out in time [40]. In this case the quiver motion only contributes as an additional cycle-averaged potential energy (the ponderomotive energy *U*_P_), which follows the slowly varying time-dependent envelope of the SPP field. As such, the ponderomotive energy transiently modifies the ionization threshold (black curve in Figure 3) and the final state energy (red curve in Figure 3). Due to energy conservation during the emission process, the pondermotive energy needs to be entered into the energy budget for the kinetic energy *E*_kin_(*t*) = *nℏω* − *eϕ* − *E*_b_ − *U*_P_(*t*), where *E*_b_ is the binding energy of the surface state, *n* is the order of the emission process, and *ℏω* is the SPP energy. As the field strength decays in time (towards the right in Figure 3), the ponderomotive energy decreases, and the emitted electron loses kinetic energy by adiabatically following the temporal change of the ponderomotive potential. Considering that the likelihood for electron emission is the highest when the field is the strongest (*t* = 0), and that the ponderomotive energy vanishes after the field has decayed (*t* = ∞), the overall change of the electron’s kinetic energy Δ*E*_kin_ amounts to

(1) $$\Delta E_{\text{kin}}=-\Delta U_{\text{P}}=-U_{\text{P}}\left(0\right)=-\frac{e^{2}|E_{\text{max}}{|}^{2}}{4m_{\text{e}}\omega^{2}}.$$

Since the equation for Δ*E*_kin_ depends linearly on the maximum intensity of the SPP field ${\left|E_{\text{max}}\right|}^{2}$ , Δ*E*_kin_ decreases linearly as the laser power is increased, and the emission spectra in Figure 2(c) must linearly shift to lower final-state energies with the aforementioned experimentally determined slope of −425 ± 13 meV/W. In our experiment we find a maximal ponderomotive energy of 148 ± 5 meV at a maximal average laser power of 349 mW.

The pondermotive energy in Eq. (1) only depends on the absolute SPP field strength, the frequency of the field, and fundamental constants. Therefore, the maximum SPP field strength can be directly inferred from the experimental estimate of the ponderomotive energy – without knowledge of any other experimental parameters. For the SPP focus in the center of the Archimedean spiral in Figure 2(b) we calculate a maximal field strength of 4.3 V/nm.

The results of our experiment can be further discussed within the terminology of the Keldysh parameter [42] and the adiabaticity parameter [10, 33] that are both useful to characterize the time-scale and the length-scale of the emission process, respectively.

The Keldysh parameter $\gamma ={\left(E_{i}/2U_{\text{P}}\right)}^{1/2}$ compares the ponderomotive energy *U*_p_ to the ionization energy *E*_i_ = *eϕ* + *E*_b_. Considering the work function of the Au(111) surface of *eϕ* = 5.3 eV [32] and the binding energy of the Au(111) Shockley surface state of *E*_b_ = 0.48 eV [43], the experimentally determined ponderomotive energy converts into a Keldysh parameter of *γ* = 4.4. Large Keldysh parameters describe multiplasmon absorption, whereas for small Keldysh parameters tunnel-ionization dominates. According to Pant and Ang [44] the critical Keldysh parameter to distinguish the two cases for electron emission from the Au surface state is *γ*_crit_ = 2.8. Our measured Keldysh parameter of 4.4 indicates multiplasmon absorption, which is consistent with the shape of the spectra in Figure 2(a) and (c).

The adiabaticity parameter *δ* = *l*_*F*_/*l*_*q*_ was introduced to describe electron emission from localized near-fields and compares the amplitude *l*_*q*_ of the quiver motion of the emitted electron to the evanescent decay length *l*_*F*_ of the electric field in the direction perpendicular to the surface. For *δ* ≫ 1 this field localization is negligible with respect to the quiver motion of the emitted electron, such that the ponderomotive interaction is dominated by the local field strength at the surface rather than the spatial field gradient. For the flat Au(111)/vacuum interface the decay length of the SPP field *l*_*F*_ = 650 nm can be easily calculated from the dielectric constants [45]. The experimentally determined maximal ponderomotive energy implies a maximal quiver amplitude of *l*_*q*_ = 0.14 nm. The combination of these two quantities allows calculating the adiabaticity parameter *δ* ≈ 4700. Clearly, we are in the limit of a non-localized field.

The knowledge of both *δ* and *γ* allows us to add numbers to the discussion of Figure 3. The electron emitted from the surface state in 4th order has a kinetic energy of ≈0.4eV in the vacuum and – neglecting ponderomotive effects – would drift only maximally 11 nm away from the surface during the presence of the ≈30fs short SPP pulse. Thus the electron almost rests in front of the surface while it performs a minimal 0.14 nm quiver motion. The quiver motion thus averages out in each optical cycle, and only the adiabatic energy loss from the temporal envelope of the SPP field remains. The measured ponderomotive energy is in this picture representative of the maximal local SPP field strength.

## 4 Conclusion

Compared to similar experiments using sharp tips [33–35], nanoparticles [46], or SPPs at prism surfaces [36–39] we obtain a much larger adiabaticity parameter as result of the small quiver motion and the slow evanescent decay of the SPP field perpendicular to the surface. Combined with a Keldysh parameter of *γ* = 4.4 this creates a rather unique situation where the interaction of the emitted electron with the strong SPP field reduces to an adiabatic energy exchange with the ponderomotive potential. Hereby, the spatiotemporal separation of the exciting laser pulse from the SPP pulse enables an independent investigation of the SPP focus, whithout the presence of a light field. Only under these particular experimental conditions can the SPP field strength be directly inferred from the measured ponderomotive energy.

The obtained experimental situation of a SPP focus within an Archimedean spiral and with a known field orientation provides the opportunity to investigate defined band structures on flat surfaces in multi-plasmon absorption. In the simplest case, this could be the Shockley surface state of the plasmonic Au(111) substrate. However, by placing materials with a different band structures in the strong-field SPP focus, our experiment offers exciting possibilities to study strong-field light–matter interactions like AC-Stark shifts [47, 48], Bloch–Siegert shifts [49], or Floquet band splitting [50–52] in strong SPP fields in a variety of materials and with spatial resolution.

## Supplementary Material

Supplementary Material Details
