# Supplementary material for: Quantitative determination of the electric field strength in a plasmon focus from ponderomotive energy shifts
Source: Nanophotonics. 2022 Aug 2;11(16):3687–94. doi: 10.1515/nanoph-2022-0284 (PMC11501193; doi:10.1515/nanoph-2022-0284)
Supplement: Supplementary file 1 — Supplementary Material Details [file j_nanoph-2022-0284_suppl.pdf]

Supplementary Material for "Quantitative Determination of the Electric Field Strength in a Plasmon Focus from Ponderomotive Energy Shifts"

Pascal Dreher[1], David Janoschka[1], Alexander Neuhaus[1], Bettina Frank[2], Harald Giessen[2], Michael Horn-von Hoegen[1], Frank-J. Meyer zu Heringdorf[1,3*]

[1] Faculty of Physics and Center for Nanointegration, Duisburg-Essen (CENIDE), University of Duisburg-Essen, 47048 Duisburg, Germany

[2] 4th Physics Institute, Research Center SCoPE, and Integrated Quantum Science and Technology Center, University of Stuttgart, 70569 Stuttgart, Germany

[3] Interdisciplinary Center for the Analytics on the Nanoscale (ICAN), 47057 Duisburg, Germany

∗ Corresponding author. E-Mail: meyerzh@uni-due.de

S1. Analysis of Spectral Features

To verify that the spectral feature close to the Fermi edge replica in Fig. 2 (a) of the manuscript is indeed caused by the Au(111) surface state, a spectral decomposition of the 5th order part of the spectrum was performed. The result is shown in Figure S1.

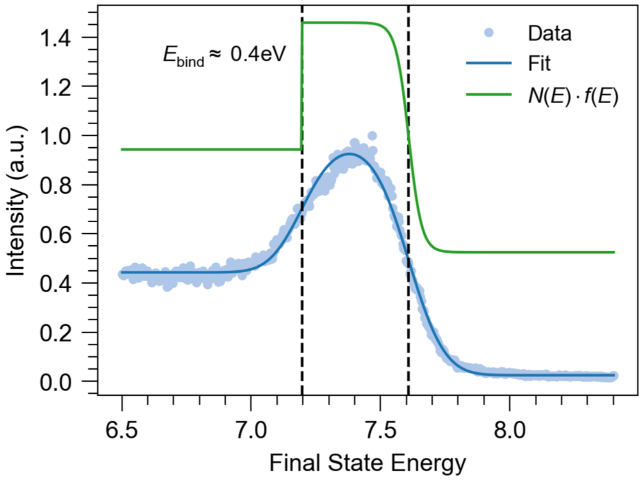

Figure S1: a linear representation of the 5th order part of the spectrum in Fig. 2 (a) of the manuscript. The experimental data is shown as blue dots, the overall fit is represented as the blue line. The green curve shows the assumed density of states $N(E) = N_{SS}(E) + N_{Au}(E)$ that is cut off by the Fermi edge at higer binding energies. The vertical dashed lines mark the energy of the surface state (left) and the position of

The same data that was used for Fig. 2 (a) is shown in Fig. S1 in a linear representation as blue dots, just that the range of final state energies is limited to the range between 6.5 and 8.4 eV. Since the surface state is a parabolically dispersing band in two dimensions, and since the data was acquired by integrating over the entire emission angle, the surface state was modeled by a step-wise density of states $N_{SS}(E) = \alpha \cdot \Theta(E - E_F - E_{bind})$ where E_F is the Fermi energy in 5th order, E_{bind} is the binding energy of the surface state, and α is a constant. On the high energy side the spectral feature is cut off by the Fermi distribution $f(E - E_F)$. We use a constant density of states $N_{Au}(E) = \beta$ to account for Au states that contribute to the emission yield but that are not part of the surface state. The limited instrumental resolution of the energy filter was accounted for by convolving the overall density of states with a Gaussian function $g(E)$. Altogether the used fit function was

$$I(E) = \left[f(E - E_\mathrm{F}) \cdot \left(N_\mathrm{SS}(E) + N_\mathrm{Au}(E)\right)\right] * g(E) + \gamma,$$

using E_F, E_bind, α, β, γ, and the width of $g(E)$ as free parameters. The width of the Fermi distribution was kept fixed at 25 meV (room temperature).

A combination of a differential evolution algorithm with Monte Carlo sampling for determination of the stability was performed. The product of the resulting overall density of states $N(E) = N_\mathrm{SS}(E) + N_\mathrm{Au}(E)$ and the Fermi distribution is plotted as a vertically shifted green line in Fig. S1, and the optimal fit is shown as a solid blue line. The fitted binding energy of 0.4 eV lies within the range of the binding energies of the Au surface state of 0.38 - 0.50 eV compiled by Woodruff et al.[1] The analysis indicates that our spectral feature is indeed explained by the Au(111) surface state.

Supplementary References

1. D. P. Woodruff, W. A. Royer and N. V. Smith, Phys Rev B **34** (2), 764-767 (1986).
